# Phenotypic H-Antigen Typing by Mass Spectrometry Combined with Genetic Typing of H Antigens, O Antigens, and Toxins by Whole-Genome Sequencing Enhances Identification of Escherichia coli Isolates

**DOI:** 10.1128/JCM.00422-16

**Published:** 2016-07-25

**Authors:** Keding Cheng, Huixia Chui, Larissa Domish, Angela Sloan, Drexler Hernandez, Stuart McCorrister, Alyssia Robinson, Matthew Walker, Lorea A. M. Peterson, Miles Majcher, Sam Ratnam, David J. M. Haldane, Sadjia Bekal, John Wylie, Linda Chui, Shaun Tyler, Bianli Xu, Aleisha Reimer, Celine Nadon, J. David Knox, Gehua Wang

**Affiliations:** a National Microbiology Laboratory, Public Health Agency of Canada, Winnipeg, MB, Canada; b Department of Human Anatomy and Cell Sciences, College of Medicine, University of Manitoba, Winnipeg, MB, Canada; c Henan Center of Disease Control and Prevention, Zhengzhou, Henan Province, China; d Newfoundland and Labrador Public Health Laboratory, St. John's, NL, Canada; e Nova Scotia Provincial Public Health Laboratory Network, Halifax, NS, Canada; f Laboratoire de Santé Publique du Québec, Sainte-Anne-de-Bellevue, QC, Canada; g Cadham Provincial Laboratory, Winnipeg, MB, Canada; h Provincial Laboratory for Public Health and Department of Laboratory Medicine and Pathology, University of Alberta, Edmonton, AB, Canada; i Department of Medical Microbiology, College of Medicine, University of Manitoba, Winnipeg, MB, Canada; Medical College of Wisconsin

## Abstract

Mass spectrometry-based phenotypic H-antigen typing (MS-H) combined with whole-genome-sequencing-based genetic identification of H antigens, O antigens, and toxins (WGS-HOT) was used to type 60 clinical Escherichia coli isolates, 43 of which were previously identified as nonmotile, H type undetermined, or O rough by serotyping or having shown discordant MS-H and serotyping results. Whole-genome sequencing confirmed that MS-H was able to provide more accurate data regarding H antigen expression than serotyping. Further, enhanced and more confident O antigen identification resulted from gene cluster based typing in combination with conventional typing based on the gene pair comprising *wzx* and *wzy* and that comprising *wzm* and *wzt*. The O antigen was identified in 94.6% of the isolates when the two genetic O typing approaches (gene pair and gene cluster) were used in conjunction, in comparison to 78.6% when the gene pair database was used alone. In addition, 98.2% of the isolates showed the existence of genes for various toxins and/or virulence factors, among which verotoxins (Shiga toxin 1 and/or Shiga toxin 2) were 100% concordant with conventional PCR based testing results. With more applications of mass spectrometry and whole-genome sequencing in clinical microbiology laboratories, this combined phenotypic and genetic typing platform (MS-H plus WGS-HOT) should be ideal for pathogenic E. coli typing.

## INTRODUCTION

Escherichia coli is a common bacterium, and pathogenic E. coli-contaminated food and/or water can cause severe health problems, such as hemolytic-uremic syndrome (HUS), in humans (1). Consequently, rapid and accurate identification and typing of E. coli are very important to those who have been affected by such contamination, particularly during E. coli outbreaks. E. coli is conventionally identified via biochemical tests (2) and typed by the serotyping of two major surface antigens, lipopolysaccharides (LPS; O antigens) and flagellar proteins (H antigens) (3). The pathogenicity of these bacteria is examined through cytotoxicity assays (4) or by checking for the presence of suspected toxins (or their related genes) by methods such as enzyme-linked immunosorbent assay or PCR, especially on common toxins such as Shiga toxin 1 (Stx_1_) or Shiga toxin 2 (Stx_2_) (4, 5). Currently, serotyping methods are not sufficiently rapid to quickly and accurately identify an unexpected E. coli strain because of the large number of possible O (O1-O188; O31, O47, O67, O72, O94, and O122 have been withdrawn) and H (H1 to H56; H13, H22, and H50 have been withdrawn) antigens (6–8). In addition, during O-antigen serotyping, rough strains often arise, likely because of unfavorable growth conditions or genetic mutations (9–11) that cause a lack of antigen-specific sugar/sugar chains on the LPS molecule. For H-antigen serotyping, clinical isolates routinely require flagellum growth induction to optimize the H-antigen–antiserum agglutination reactions, a very time-consuming process (8). Even with motility induction, many isolates are still designated H nonmotile (NM) or undetermined after repeated serotyping (12). Over the past few years, we have focused our efforts on developing a new method for more accurate and rapid typing of E. coli H antigens by using mass spectrometry (8, 12). This H typing method was named MS-H (8), and validation using clinical isolates proved that it displayed higher speed, better sensitivity, and better specificity than conventional serotyping (12–14). In this study, we formally applied whole-genome sequencing (WGS) to the MS-H typing process, with a focus on resolving clinical isolates that could not be assigned an H type by conventional serotyping and on those isolates whose H serotypes were in disagreement with MS-H-assigned types. The H antigens, O antigens, and toxin genes of these isolates (representing NM, H-undetermined [Hund], and rough strains) were all analyzed. We termed the approach MS-H plus WGS-HOT (mass spectrometry-based H-antigen typing plus WGS-based H-antigen, O-antigen, and toxin identification). We proposed that if the approach was successful, a genotype- and phenotype-based method for targeted identification and typing of the important biomarkers of pathogenic E. coli (H antigens, O antigens, and toxins) could be created and used in clinical microbiology laboratories.

## MATERIALS AND METHODS

### E. coli strains.

All of the E. coli reference strains in this study were obtained from the ISO-certified Enterics Reference Centre at the National Microbiology Laboratory, Public Health Agency of Canada. Sixty clinical strains used in this study for WGS were among 219 clinical isolates received from five Canadian provincial laboratories for public health (Alberta, Manitoba, Quebec, Newfoundland, and Nova Scotia) for routine serotyping and MS-H typing.

### H- and O-antigen serotyping.

For H-antigen serotyping, an ISO-certified method was used (8). Motility induction was performed for a maximum of 2 weeks. Motile strains underwent agglutination reactions with multivalent antiserum pools and formalin-treated bacterial culture. Monovalent antisera were used to determine the serotype of a particular isolate. For O typing, no motility induction was needed but a similar strategy was used. Agglutination reactions were performed with multivalent antiserum pools, and monovalent antisera were then used to determine the O serotype of a particular isolate.

### Shiga toxin (verotoxin) testing by PCR.

The majority of the clinical isolates were found to contain *stx*_1_ and *stx*_2_ (the two most common E. coli toxin genes) by provincial laboratories by various PCR methods and reported as either verotoxin positive or *stx*_1_/*stx*_2_ positive. Some provincial laboratories also tested for *hlyA* (encoding hemolysin A) and *eae* (encoding intimin). The *stx*_1_, *stx*_2_, and *hlyA* genes of nonreported reference strains and clinical isolates at the National Microbiology Laboratory of Canada were amplified by a previously established protocol (15), with some modifications. In brief, four colonies resulting from overnight growth of freshly cultured cells were mixed with 1 ml of deionized water and boiled for 10 min. The mixture was then centrifuged at 15,000 × *g* for 10 min, and 1.5 μl of supernatant was mixed with 50 μl of a PCR mixture containing 1× Mastermix (Bio-Rad) and 5 μM primers targeting the *stx*_1_, *stx*_2_, or *hlyA* gene (15). PCRs were performed under the following conditions: initial denaturation at 98°C for 5 min; 35 cycles of denaturation at 98°C for 15 s, annealing at 60°C for 20 s, and extension at 72°C for 20 s; and a final extension at 72°C for 8 min.

### MS-H typing.

Motility induction was not performed. A 10-μl loop of the overnight culture of each clinical isolate was used for flagellum extraction. Each culture was mixed in water, and the flagella were sheared from the cell body by vortexing for 60 s (8). The cell bodies were centrifuged at 16,000 × *g* for 20 min and the supernatant (containing detached flagella) was applied to a syringe filter (8, 12–14). The flagella were then washed and digested on the filter for 2 h at 37°C. The tryptic digests were flushed out of the filter with water and tested on the Orbitrap-XL (ThermoFisher) liquid chromatography-tandem mass spectrometry (LC-MS/MS) system. MS data were searched against a curated database containing E. coli flagellin sequences by using a Mascot search engine to obtain emPAI (exponentially modified protein abundance index) values (calculated as 10 to the power of [the number of observed peptides divided by the number of observable peptides] minus 1) (12). The following rules were used to assign an H type. (i) A minimum emPAI value of 1 was necessary. (ii) The top hit should have an emPAI value at least twice that of the previous adjacent blank run when the emPAI value of the previous blank run exceeded 1 because of carryover. (iii) Repeated jigsaw cleanups and LC-MS/MS analyses of samples were performed after blank runs showing emPAI values of >1. (iv) If the adjacent previous blank run had no significant flagellin identified (fewer than two specific peptides) and the emPAI value of the subsequent sample run was 0.10 to 0.99 because of low flagellum production from “sluggish” isolates, repeat testing should be performed with a larger sample amount to obtain an emPAI value of ≥1 (13, 14).

### WGS-HOT.

**FIG 1:**
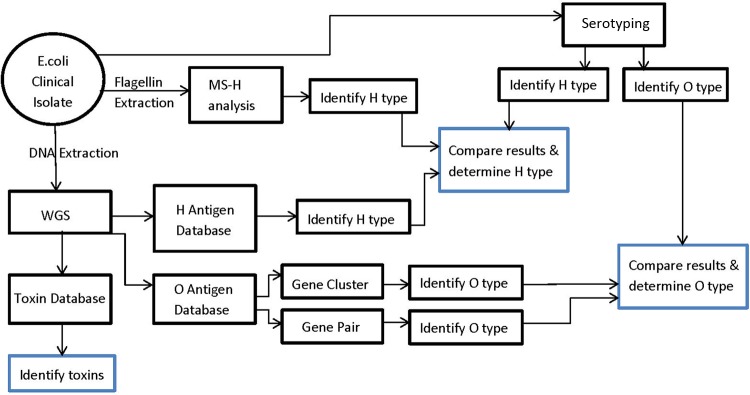
Work flow of MS-H plus WGS-HOT.

The work flow of WGS-HOT, together with MS-H typing, is shown in Fig. 1, and detailed methods are described in the supplemental material. In brief, two to four colonies of overnight E. coli culture were collected and the genomic DNA was extracted by using Epicentre Metagenomic DNA Isolation kits (Mandel Canada). The DNA quality was checked by visualizing electrophoresis products on a 1% agarose gel and quantified with a Qubit DNA quantification system (Invitrogen). At concentrations between 10 and 50 ng/μl, WGS was performed with a Nextera XT DNA Sample Preparation kit (Illumina) and data acquired by 300-bp paired-end sequencing on the Illumina MiSeq with the MiSeq Reagent kit V2 (600 cycles). An *in silico* analysis of the sequences of each isolate was performed, where data were assembled into contigs with SPAdes Assembler (v2.5.1). Galaxy software was used to search for H antigen, O antigen, and toxin genes against individually created curated databases.

For a proof-of-principle demonstration, MS-H typing and WGS-HOT were initially performed with well-known E. coli reference strains such as EDL933 (ATCC 43895; an Stx_1_- and Stx_2_-producing strain) and K-12 (MG1655, ATCC 47076; a nonpathogenic rough strain), as well as those examined in our earlier studies (13, 15). Seventeen randomly chosen clinical isolates whose serotypes and MS-H types were in agreement were also tested to verify the method, but because of the high cost of performing WGS, WGS-HOT was focused mainly on problematic isolates (i.e., Hund, NM, and O rough isolates and those whose MS-H and serotyping results did not agree) (12–14). All databases were compiled in accordance with the strategy used for MS-H typing (8, 12–14). The H-antigen and toxin gene databases were created by using all of the reported E. coli flagellin and toxin gene sequences available in the NCBI (National Center for Biotechnology Information) data bank (8, 16–18). For a list of the toxins and virulence factors, see Table S1 in the supplemental material. The O-antigen database was initially composed of only the gene pair comprising *wzx* and *wzy* or that comprising *wzm* and *wzt*, as these genes had been reported to be very specific for most O antigens (6, 7). However, after several trials, it was determined that the creation of another database comprising the entire O-antigen gene cluster, including every enzyme/protein gene involved in O-antigen synthesis and transport (19–22), was necessary. All of the genes were annotated in order to adhere to the Galaxy search platform, which was updated once every few months (23). Randomly chosen WGS data on reference strains and clinical isolates stemming from five batches of WGS-HOT analysis were also checked by using the publicly available SeroTypeFinder and VirulenceFinder platforms from the Center for Genomic Epidemiology (CGE) (24).

## RESULTS

As shown in Table 1, a total of 12 reference strains were tested. MS-H plus WGS-HOT identified all of the H antigens, O antigens, and common toxins accordingly. Notably, one reference strain (E32511) identified as NM by serotyping and MS-H (8, 14, 15) still showed an H type by WGS. This finding illustrates the important point that the genetic existence of an H antigen does not guarantee its expression, and therefore, phenotypic identification through serotyping or MS-H is necessary to confirm H-antigen protein expression (25). For O antigens, two curated databases (gene pair and gene cluster) were used. The O types of 11/11 strains were correctly identified by using the gene pair database, although a range of percent coverage was needed in the database search (26). Interestingly, K-12, a well-known O rough strain with no O-antigen serotype, also showed an O-type-specific gene. By using the gene cluster database, all O antigens were typed correctly when the housekeeping gene *gne* (19–22) was ruled out for strain 90-2380. This nonspecific gene showed the same confidence as *wbdO*, a nonhousekeeping gene (19). Interestingly, searches against the O-antigen gene cluster database often gave top hits different from those obtained with the gene pair database, although the two databases overlapped. This indicated that other members of the gene cluster are complementary to the gene pair comprising *wzx* and *wzy* or that comprising *wzm* and *wzt* and could be useful for O-antigen identification. The WGS approach identified additional toxin genes and virulence factors beyond the *stx*_1_ and/or *stx*_2_ genes (15).

**TABLE 1:** Results of MS-H plus WGS-HOT analysis of E. coli reference strains^*a*^

Strain	Serotype	MS-H	H-antigen gene database	O-antigen gene database					Toxin and virulence factor gene database^*k*^
				Gene cluster	Gene pair 90%	Gene pair 80%	Gene pair 70%	Gene pair 60%	
E32511^*b*^	O157:HNM^*d*^	NM^*d*^	H7	O157-fcl	O157-wzx	NA^*i*^	NA	NA	Stx2, HlyA, intimin, 14 others
EDL933^*b*^	O157:H7	H7^*e*^	H7	O157-wbdQ	NO^*j*^	NO	O157-wzx	NA	Stx1, Stx2, HlyA, intimin, 15 others
90-2380^*b*^	O157:H7	H7^*e*^	H7	O157-wbdO O86-gne^*h*^	O157-wzx	NA	NA	NA	Stx2, HlyA, 12 others
H19^*b*^	O26:H11	H11^*f*^	H11	O26-wbuA	O26-wzy	NA	NA	NA	Stx1, HlyA, intimin, 14 others
12-0721^*c*^	O26: H11	H11^*e*^	H11	O26-wzy	O26-wzy	NA	NA	NA	HlyA, intimin, 14 others
B2F1^*b*^	O91:H21	H21^*f*^	H21	O91-wbsB	O91-wzx	NA	NA	NA	Stx2, HlyA, 7 others
86-704^*b*^	O15:H27	H27^*f*^	H27	O15-wzy	O15-wzy	NA	NA	NA	Stx2, HlyA, 7 others
H.I.8^*b*^	O128:B1^*g*^	H2^*f*^	H2	O128-wbsI	NO	O128-wzx	NA	NA	Stx2, HlyA, intimin, 7 others
12-2628^*c*^	O103:H25	H25^*e*^	H25	O103-wbtF	O103-wzx	NA	NA	NA	Stx2, HlyA, intimin, 10 others
12-0747^*c*^	O25:H4	H4^*e*^	H4	O25-rmlA	NO	NO	NO	O25-wzy	6 others
09-0414^*c*^	O104:H7	H7^*e*^	H7	O104-manC	NO	NO	NO	O104-wzx	HlyA, subtilase, 8 others
K-12^*c*^	Orough:H48	H48^*e*^	H48	O16-wzy	O16 wzy	NA	NA	NA	3 others

a *E. coli* reference strains were identified by serotyping and MS-H and further analyzed by WGS-HOT using curated databases. Parameters were set at a 90% minimum coverage cutoff for the H-antigen, O-antigen gene cluster, and toxin database searches, while various degrees of coverage (60 to 90%) were set for the O-antigen gene pair database.

b Literature has shown that these strains are known to have common toxin genes such as *stx*_1_, *stx*_2_, and *hlyA* (15).

c Serotypes were reported earlier (12), and *stx*_1_, *stx*_2_, and *hlyA* genes were confirmed by PCR.

d NM, nonmotile.

e H types confirmed by LC-MS/MS (12).

f H types confirmed by matrix-assisted laser desorption ionization–time of flight mass fingerprinting (16).

g Serotype originally identified without H type designation (15).

h Nonspecific gene based on reference 19.

i NA, not applicable.

j NO, not obtainable.

k Only important toxins (15) were mentioned, and the rest were categorized as “others” (Stx1, Shiga-like toxin 1; Stx2, Shiga-like toxin 2; HlyA, hemolysin A).

Table S2 in the supplemental material shows the results of WGS-HOT performed with 17 randomly selected clinical isolates whose MS-H types and serotypes were in agreement. Markedly, the WGS results match the phenotypic H typing results in all 17 cases. Of another 17 isolates designated NM by serotyping, MS-H identified 11 as flagellum positive (64.7%), with 10/11 (90.9%) H types in agreement with the WGS outcome (see Table S3 in the supplemental material). These results indicate that MS-H typing is more sensitive than serotyping for phenotypic H-antigen identification. Similar to reference strain E32511, H types were still obtained by WGS for the remaining six NM isolates that neither serotyping nor MS-H could identify. This further confirmed that H antigens characterized by WGS do not necessarily represent phenotypic H types, as genes responsible for flagellum production are not always expressed (27).

WGS-HOT tests of five Hund isolates (see Table S4 in the supplemental material) indicated clear H types by MS-H, which were confirmed by WGS. This observation confirmed previous findings indicating that MS-H is more specific than traditional serotyping for identifying H types at the protein sequence level (12). Moreover, for 21 isolates with discordant serotypes and MS-H types, the WGS H types were in better agreement with MS-H (12/21; 57.1%) than with serotyping (4/21; 19.0%) (see Table S5 in the supplemental material). Notably, WGS did not provide results concordant with the phenotypic data in four cases and could not designate an H type for one isolate.

Table 2 summarizes the results of the three H-typing platforms (i.e., serotyping, MS-H, and WGS) for all 60 of the clinical isolates under examination. These data suggest that MS-H typing is more sensitive and accurate than traditional serotyping for NM isolates, Hund strains, and discordant isolates whose serotyping and MS-H results do not agree.

**TABLE 2:** Test summary and comparison of E. coli isolates by serotyping, MS-H typing, and WGS based H typing^*a*^

Serotyping category (no. of isolates)	No. of MS-H types obtained	No. of serotypes concordant with WGS assigned H types	No. of MS-H types concordant with WGS assigned H types	No. of WGS assigned H types in disagreement with serotype and MS-H types^*b*^
Serotype concordant with MS-H type^*b*^ (17)	17	17	17	0
NM isolates (17)	0	NA^*c*^	NA	6
	11	NA	10	1
Undetermined H type (5)	5	NA	5	0
Serotype different from MS-H type (21)	21	4	12	5^*d*^

a Tests of all 60 isolates were summarized.

b The isolates tested were randomly chosen from 219 clinical isolates.

c NA, not applicable.

d There was no WGS result for 1 isolate; WGS was performed, but no H type result was obtained.

The results of O-antigen WGS analyses of 56 clinical isolates having clear O types by O-antigen serotyping (no O rough strains included; see Tables S2 to S5 in the supplemental material) are summarized in Table 3. When the gene pair database was used for O-antigen identification and typing, most of the top hits (44/56; 78.6%) matched the corresponding serotype designations. The remaining 12/56 isolates (21.4%) were identified as having O types different from those designated by serotyping (2/56, 3.6%), showed no O type at all although a different coverage threshold was applied (5/56, 8.9%), or showed multiple hits with similar confidence (5/56, 8.9%). These multiple hits with high sequence similarity were also reported by others (20, 21, 24, 28); for example, O123/O186, O77/O17, and O117/O107. When the gene cluster database was used, all of the isolates were given O-antigen designations, with 45/56 isolates (80.4%) matching those identified by serotyping when housekeeping genes (e.g., *gnd*, *gne*, *ugd*, *wzz*, *galF*, and *hisI*) were ruled out (19–22, 28, 29). Similar to the gene pair database, 11/56 isolates (19.6%) either showed multiple top hits (7/56, 12.5%) with the same confidence score or hits different from the serotyping results (4/56, 7.1%). Interestingly, the top hits of three isolates were housekeeping genes (*ugd*, *galF*) but corresponded to serotypes found during the gene cluster database search. Similar to the results obtained with reference strains, a large number (all but two isolates) of top hits from the gene cluster database were not based on the gene pair comprising *wzx* and *wzy* or that comprising *wzm* and *wzt*. This phenomenon was exemplified when an O type was revealed for two unidentified isolates (14-8954 and 14-7998) when the gene pair database was applied alone. This observation confirmed that other genes could be useful for O-antigen identification. The combined results of the two O-antigen databases proved complementary, as 94.6% of the O antigens were in agreement with serotyping, compared to 78.6% with the gene pair database alone.

**TABLE 3:** Results of MS-H plus WGS-HOT analysis of the O-antigen and toxin genes of 56 clinical E. coli strains

Antigen ID	No. of isolates/total (%) in O-antigen gene database			No. of isolates/total (%) in toxin/virulence factor gene database^*b*^
	Gene pair^*a*^	Gene cluster	Both databases^*a*^	
Identical to serotype	44/56 (78.6)^*c*^	45/56 (80.4)^*e*^	53/56 (94.6)	55/56 (98.2)^*g*^
Inconsistent with serotype^*e*^	12/56 (21.4)^*d*^	11/56 (19.6)^*f*^	3/56 (5.4)	1/56 (1.8)^*h*^

a Cumulative results over all coverage levels (60 to 90%).

b Only toxin detection results obtained by the WGS method are shown.

c Multiple hits involving similar O types (two isolates with O123 and O186; two isolates with O77, O17, O44, and O73; and one isolate with O8 and O174) were not included.

d Five of the 12 isolates had no hit at all, another 5 had two or more hits (two isolates with O123 and O186; two isolates with O77, O17, O44, and O73; and one isolate with O8 and O174 for *wzm*), and two had hits different from the serotype.

e Common genes (*gne*, *gnd*, *wzz*, *hisI*, *galF*) were excluded when multiple hits were observed on the basis of current literature reports but were used when a single hit matched the serotype; multiple hits involving similar O types (one isolate with O123 and O186; one isolate with O117 and O107; one isolate with O77, O17, O44, and O73; and one isolate with O8 and O174 for *wzm*) were not included.

f Seven isolates had multiple “top hits” that were difficult to elucidate on the basis of current literature reports, while four isolates showed hits different from the serotyping results.

g Showed presence of toxin/virulence factor genes.

h Did not show presence of toxin/virulence factor genes.

Four (6.7%) of the 60 clinical isolates were identified as O rough (see Table S6 in the supplemental material). When samples were analyzed by using the gene cluster database, O-antigen-related genes were identified in all four isolates, though all were housekeeping genes (*galF*, *gne*, or *ugd*) (19–22). A gene pair database search provided an O type for only one isolate (11-5490), while WGS analysis performed twice on strain 14-6184 with separate sample preparations showed the same result with no specific O-type-related gene identified.

Since two housekeeping genes, *galF* and *ugd*, showed some specificity for O-antigen identification, these two genes were compared across three O types (see Table S7 in the supplemental material). The genes did show differences in sequence length (*galF*, 221 to 784 nucleotides; *ugd*, 1,128 to 1,167 nucleotides) and nucleic acid composition (*galF*, 80.96 to 96.08% similarity; *ugd*, 96.66 to 96.92% similarity).

WGS analysis based on the toxin gene database showed the existence of various toxin genes/virulence factors in 59/60 (98.3%) clinical isolates (Table 3; see Table S6 in the supplemental material). The *stx*_1_, *stx*_2_, and *hlyA* genes were identified in the majority of the PCR-confirmed cases (the *hlyA* gene was identified in all but four isolates). Additional Shiga toxin subtypes 2c, 2d, and 2f and another common toxin, intimin (*eae*), not routinely tested for by all provincial clinical microbiology laboratories, were found. Subtilase, representing a family of E. coli toxins linked to HUS (30), was detected in one reference strain (09-1414) and two clinical isolates (11-6008 and 14-6184). Reference strain EDL933 and three clinical isolates (14-4602, 14-4603, and 14-4604) were found to have both the *stx*_1_ and *stx*_2_ genes by WGS. Many virulence factors were also identified by WGS, some of which may not be directly involved in the pathogenicity of the cells. For example, three virulence factors were identified for nonpathogenic strain K-12. Similarly, if only well-verified E. coli toxins (*stx*_1_, *stx*_2_, *hlyA*, intimin, subtilase, etc.) were considered, some clinical isolates might be regarded as nonpathogenic.

The identifications of common toxins (*stx*_1_, *stx*_2_, and *hlyA*) by WGS-HOT and the CGE platforms SeroTypeFinder and VirulenceFinder were very similar (data not shown). Of the 20 strains analyzed for H antigens, only 1 was incorrectly identified by Galaxy (and accurately identified by SerotypeFinder). The O antigen of four clinical isolates was correctly determined by Galaxy (two were based on the gene cluster database only) but not SerotypeFinder, even at the lowest search threshold (see Table S8 in the supplemental material) (24). Neither WGS-HOT nor CGE software was able to identify two O rough isolates.

The performance of the three platforms (serotyping, MS-H typing, and WGS-HOT) is summarized in Table 4.

**TABLE 4:** Comparison of the E. coli serotyping, MS-H typing, and WGS-HOT platforms^*a*^

Parameter	Serotyping (O and H antigens)	MS-H typing	WGS-HOT
Capability	Detects phenotypic presence of O and H antigens	Detects phenotypic presence of H antigens	Detects genetic presence of H antigens, O antigens, toxins
Diagnostic sensitivity	High^*b*^	High	High
Diagnostic specificity	High	High	High
Analytical sensitivity	Loop culture	Subsingle colony	Multiple colonies
Analytical specificity	Antibody specific	Amino acid sequence specific	Nucleic acid sequence specific
Readout	Subjective agglutination titer observation; from − to ++++; multiple steps	emPAI values or sequence coverage; one step	Sequence coverage, divergence, depth, others; one step
Motility induction	Routinely required; 2–14 days	Not required; 0 days	Not required; 0 days
Time to get result	7–14 days	4 h	7 days
Rough strains	Impossible; 0.0% identification	Possible; up to 100.0% identification	Not applicable (not phenotypic)
Repeatability	Good^*c*^	Good	Good
Reproducibility	Good	Good	Not thoroughly studied
Throughput	Manually, 20 isolates/day with multiple reactions/isolate in multiple days	Mainly automatic, 42 isolates/wk with single detection/isolate	Mainly automatic, 24 isolates/wk with single detection/isolate
Sample preparation	Multiple steps	Single step	Multiple steps
Cost of consumables and labor used	$12/strain; up to days of labor	$9/strain; 0.5 h of labor	$50/strain; half day of labor
System suitability	Reference laboratories with antiserum or antibody production	Institutions or service laboratories with MS capability	Institutions or service laboratories with WGS capability

a Based on literature reports and our experience in the past and in the present study.

b High, >80%.

c Good, >80%.

## DISCUSSION

This study represents an expansion of MS-H of E. coli (8, 12–14) to MS-H plus WGS-HOT. Initially, MS-H was designed to reduce the duration of outbreak investigations by alleviating the lengthy process of traditional H serotyping. Serotyping is often time-consuming because of the necessary procedure of motility induction. With MS-H, motility induction can be avoided and H types can be obtained after overnight culture. MS-H can also be used to identify isolates shown to be problematic for serotyping (e.g., Hund or O rough strains) because of a variety of factors, such as low-quality antisera and autoagglutination. WGS was originally used to confirm the results of H serotyping and MS-H, as PCR could not obtain all of the H-antigen sequences easily or quickly because of the necessity of using different primer sets (12). We believe that combining MS-H and WGS will dramatically reduce the amount of time required to identify clinical E. coli H antigens, which will certainly prove useful in outbreak situations.

With a focus on problematic clinical isolates such as serotyping-designated Hund, NM, and rough strains, as well as those with discordant serotyping and MS-H results, this study demonstrated that MS-H is a more sensitive and specific method than serotyping because of its molecular-level identification. Essentially any tryptic peptide that can be ionized in an MS system is identifiable, a stark contrast to the limited number of antigenic epitopes available during serotyping. In addition, some H types (e.g., types H11 and H21 and types H4 and H17) are difficult to differentiate by serotyping, as their flagellar sequences are very similar (8, 12, 14). As MS-H can obtain virtually full sequence coverage of flagellar proteins, differentiation of similar strains does not pose a problem (8, 14). And because MS-H is a phenotypic identification platform, the method can distinguish motile from NM strains. Although WGS analysis also provides H type information, the method is not phenotypic and therefore cannot differentiate between motile and NM strains. The MS-H plus WGS method should be a more confident approach for H-antigen typing, with respect to both phenotypic and genetic identification.

The lack of agreement of the H types of a small number of isolates across the three platforms may be due to factors such as sample preparation during WGS, antibody quality during serotyping, and the motility status of strains at the time of serotyping or MS-H typing (8, 12–14, 24). On the basis of current data, if both MS-H and WGS are employed and different H types result, the MS-H result should be considered correct, as it reflects the phenotypic existence of the H antigen. To confirm the result, the MS-H procedure can be easily repeated (8, 12–14).

After addressing the issues associated with traditional H serotyping, it became obvious that platforms such WGS could be used to address problems arising with O-antigen serotyping as well. Although O serotyping is faster than H serotyping because there is no need for motility induction, there are many more E. coli O antigens. Serotyping of each of the 182 O antigens is a tedious and complicated procedure, and high-quality antisera remain a necessity. Because O antigens are not proteins but LPS involving various proteins/enzymes to synthesize and transport molecules, molecular approaches such as WGS are ideal for identifying unknown O antigens. Current literature reports indicate that the gene pair comprising *wzx* and *wzy* or that comprising *wzm* and *wzt* is the best candidate for resolving E. coli O antigens (6, 7, 24). Notably, these gene pairs do not include the O14 and O57 antigens, and as a result, they cannot be typed in this manner (24). This study has demonstrated that other O-antigen gene cluster members involved in O-antigen synthesis and transport can be used to complement the gene pair comprising *wzx* and *wzy* or that comprising *wzm* and *wzt* in the database, ultimately ensuring more confident identification. While using the gene cluster database, it also became apparent that some housekeeping genes (e.g., *galF* and *gnd*) showed specificity for some O antigens. This represents the first observation regarding the role of housekeeping genes in O-antigen identification. Although we do recommend keeping housekeeping genes in database searches, a very cautious procedure should be used on the basis of current data collected for E. coli O-antigen typing. First, if a nonhousekeeping gene is identified, it will be given priority over housekeeping genes, and a housekeeping gene that indicates a different O type from the nonhousekeeping gene will be ignored. Second, if a housekeeping gene is the only gene identified in the data output, then different parameters should be used to research the database for a nonhousekeeping gene (e.g., by using a different coverage threshold, as shown by Joensen et al. [24] with SeroTypeFinder). Third, if a housekeeping gene remains the only hit after a second database search with altered parameters, a repeat of the test involving sample preparation and a rerun on the genomic sequencer should be performed to confirm the result.

Incomplete expression of O antigens by O rough isolates is a well-observed phenomenon (9–11). Literature reports have shown a low rate of O-antigen-specific gene identification, likely because of genetic mutations in O-antigen-related genes (24). It will therefore be a challenge to provide O types for all of the rough strains confidently. Further study is required in this area.

Regarding E. coli toxin identification, WGS has proven to be a very powerful platform, particularly for clinical microbiology laboratories that perform routine testing for common toxins such as Shiga toxins, hemolysin, and intimin. Because of the large number of known and unknown toxins (and their related subtypes), the use of traditional identification methods such as PCR is challenging because of the requirement for numerous primer sets and optimizing conditions. Thus far, the strategy of clinical microbiology laboratories has been to identify only common toxins (e.g., Stx_1_, Stx_2_, hemolysin A, and intimin), which means that other severe toxins (e.g., subtilase) are going undetected. We recommend that WGS be used to identify the genetic existence of toxins and virulence factors in E. coli outbreak situations, before epidemiological data and other testing results are obtained to finalize their phenotypic presence and potential pathogenicity.

In this study, we found that the Galaxy database search platform gave results very similar to those obtained with web-based SerotypeFinder and VirulenceFinder for H-antigen and toxin identification, but differences were seen regarding O-antigen typing. Galaxy appears more sensitive, possibly because of differences in the sequence assembly algorithm or other search engine software parameters. Employing the gene cluster database did help increase the number of O antigens identified with Galaxy, but SerotypeFinder could only employ the gene pair database as a default. We feel that the gene cluster database is very useful for O-antigen typing and should be integrated into O-antigen typing at the molecular level.

In summary, we believe that the MS-H plus WGS-HOT platform, involving phenotypic and genomic H-antigen typing to differentiate motile and NM strains, in combination with genomic typing of O antigens and toxins by WGS, will provide an ideal platform for pathogenic E. coli characterization. With the growing familiarity of MS and WGS techniques; their excellent sensitivity and quick turnaround time, user-friendly hardware, mature databases, and better bioinformatic tools; and the lower cost of reagents, this platform should be very useful in the rapid and accurate identification and typing of pathogenic E. coli.

## Supplementary Material

Supplemental material
