# Supplementary material for: Phenotypic H-Antigen Typing by Mass Spectrometry Combined with Genetic Typing of H Antigens, O Antigens, and Toxins by Whole-Genome Sequencing Enhances Identification of Escherichia coli Isolates
Source: J Clin Microbiol. 2016 Jul 25;54(8):2162–8. doi: 10.1128/JCM.00422-16 (PMC4963523; doi:10.1128/JCM.00422-16)
Supplement: Supplemental material [file supp_54_8_2162__index.html]

Supplemental material 

# Phenotypic H-Antigen Typing by Mass Spectrometry Combined with Genetic Typing of H Antigens, O Antigens, and Toxins by Whole-Genome Sequencing Enhances Identification of Escherichia coli Isolates

## Supplemental material

**Files in this Data Supplement:**

- Supplemental file 1 -

  Tables S1 (List of toxins and virulence factors used in the *E. coli* toxin database), S2 (Results of MS-H plus WGS-HOT analysis of 17 *E. coli* clinical strains whose serotyping- and MS-H-designated H types were in agreement), S3 (Results of MS-H plus WGS-HOT analysis of 17 *E. coli* clinical strains designated "non-motile" NM by serotyping), S4 (Results of MS-H plus WGS-HOT analysis of five *E. coli* clinical strains designated ?H undetermined? by serotyping), S5 (Results of MS-H plus WGS-HOT analysis of 21 *E. coli* clinical strains whose serotyping- and MS-H-designated H types were not in agreement), S6 (Results of MS-H plus WGS-HOT analysis of *E. coli* clinical strains designated ?O rough? by serotyping), S7 (Sequence comparison of two housekeeping genes, *galF* and *ugd*), and S8 (Comparison of H typing results using the WGS-HOT and SeroTypeFinder platforms)

  PDF, 711K
- Supplemental file 2 -

  Supplemental text

  PDF, 276K
