# Supplementary material for: Phenotypic H-Antigen Typing by Mass Spectrometry Combined with Genetic Typing of H Antigens, O Antigens, and Toxins by Whole-Genome Sequencing Enhances Identification of Escherichia coli Isolates
Source: J Clin Microbiol. 2016 Jul 25;54(8):2162–8. doi: 10.1128/JCM.00422-16 (PMC4963523; doi:10.1128/JCM.00422-16)
Supplement: Supplemental material [file JCM.00422-16_zjm999095110so1.pdf]

Supplementary Table 1. List of toxins and virulence factors used in the *E. coli* toxin database

**Toxin database description**	**Variation number***
Shiga toxin 1 subunit A	3
Shiga toxin 2 subunit A	13
Shiga toxin 1 subunit B	3
Shiga toxin 2 subunit B	13
Intimin eae alpha-1	2
Intimin eae alpha-2	3
Intimin eae beta-1	6
Intimin-eae-xiR-beta2B	2
Intimin-eae-delta	1
Intimin-eae-gamma1	5
Intimin-eae-theta	3
Intimin-eae-epsilon1	2
Intimin-eae-epsilon-2	3
Initimin-zeta	4
Intimin-eae-eta	1
Intimin-eae-eta2	1
Intimin-eae-iota1	3
Intimin-eae-iota2	1
Intimin-eae-lambda	3
Intimin-eae-mu	3
Intimin-eae-nu	1
Intimin-eae-xi	1
Hemolysin-hlyA	5
Hemolysin-ehxA	7
Hemolysin-ehylA	1
Hemolysin-sheA	2
Hemolysin-E-sheA	1
Subtilase subunit A	3
Subtilase subunit B	1
Heat-stable enterotoxin 1	1
Major subunit of bundle-forming pili	1
Colicin B	1

Cloacin	1
Endonuclease colicin E2	1
Colonization factor antigen I	1
Cytotoxic necrotizing factor	1
Longus type IV pilus subunit	1
Intimin	1
Serine protease autotransporters of enterobacteriaceae SPATE	1
EHEC factor for adherence	1
Enterohemolysin	1
Serine protease autotransporters of enterobacteriaceae	1
Type III secretion system	1
Secreted protein B	1
Serine protease autotransporters of enterobacteriaceae	1
Type II secretion system	1
Putative exoprotein precursor	1
Type II secretion protein	1
Subunit A of F17 fimbrial protein	1
Involved in biogenesis of K99/F5 fimbriae	1
Fimbrial adhesion AC precursor	1
Mature fim41a/F41 protein	1
Avian E. coli hemolysin	1
Adherence protein	1
Ivasion protein shigella flexneri	1
Invasion plasmid antigen	1
Siderophore receptor	1
Enterobactin siderophore receptor protein	1
Increased serum survival	1
Plasmid-encoded catalase peroxidase	1
Longus type IV pilus	1
Long polar fimbriae	1
Heat-labile enterotoxin A subunit	1
Microcin H47 part of colicin H	1
MchC protein	1
ABC transporter protein MchF	1
Microcin M part of colicin H	1

Diffuse adherence fibrillary adhesion gene	1
Non-LEE-encoded effector A	1
Non-LEE-encoded efector B	1
Non-LEE-encoded efector C	1
EPEC adherence factor	1
Autotransporter enterotoxin	1
Serine protease autotransporters of enterobacteriaceae	1
P-related fimbrial regulatory gene	1
Serine protease autotransporters of enterobateriaceae	1
Serine protease autotransporters of enterobateriaceae	1
Plasmid-encoded enterotoxin	1
Serine protease autotransporters of enterobateriaceae	1
S-fimbrial minor subunit	1
Heat-stable enterotoxin ST-Ia	1
Heat-stable enterotoxin II	1
Shiga-like toxin 1 A-subunit	1
Shiga-like toxin 1 B-subunit	1
Shiga toxin 2 subunit A	1
Shiga toxin 2 subunit B	1
Subtilase toxin subunit	1
STEC autoagglutinating adhesion	1
Tir cytoskeleton coupling protein	1
Translocated intimin receptor protein	1
Toxin B	1
Serine protease autotransporters of enterobateriaceae	1
Serine protease autotransporters of enterobateriaceae	1
virF transcriptional activator	1

* Variation number means different entries with separate **GI (**GenInfo Identifier; NCBI**)** numbers for the toxin/virulence factor.

Supplementary Table 2 Results of MS-H plus WGS-HOT analysis on 17 *E. coli* clinical strains whose serotyping- and MS-H-designated H types were in agreement

Strain	Serotype	MS-H	H Antigen Gene Database	O antigen Gene Database					Toxin Gene Database search$^{A, B}$	Non-WGS based toxin detection
				Gene Cluster	Gene Pair 90%	Gene Pair 80%	Gene Pair 70%	Gene Pair 60%		
14-4150	O181:H4	H4	H4	O181-fnlA	NR	NR	O181-wzx	N/A	hlyA and 6 others	No common toxin detected
14-5078	O2:H6	H6	H6	O2-wegQ	O2-wzx	N/A	N/A	N/A	hlyA and 6 others	No common toxin detected
14-7221	O157:H7	H7	H7	O157-per	NR	O157-wzx	N/A	N/A	stx1, stx2, hlyA, intimin, and 9 others	stx1, stx2, intimin, hlyA
11-3580	O108:H11	H11	H11	O108-ugd*	NR	O108-wzy	N/A	N/A	stx2, hlyA, and 3 others	stx2, intimin, hlyA
11-3225	O86:H18	H18	H18	O86-gnd*	NR	NR	NR	O86-wzy	5 others	No common toxin detected
11-4968	O123:H2	H2	H2	O123-ugd*	O123-wzx<br>O186-wzx	N/A	N/A	N/A	stx1, hlyA, intimin, and 9 others	Submitted by province as VT+
14-7998	O117:H2	H2	H2	O117-galE	NR	NR	NR	NR	5 others	No common toxin detected
11-5591	O45:H2	H2	H2	O45-wbhU	O45-wzx	N/A	N/A	N/A	stx1, hlyA, intimin, and 4 others	stx1, eae, hlyA
11-7284	O27:H12	H12	H12	O27-wejx	O27-wzx	N/A	N/A	N/A	2 others	No common toxin detected
11-7286	O75:H25	H25	H25	O75-rmlC<br>O107-rmlB<br>O34-rmlB<br>O6-wzz*	O75-wzx	N/A	N/A	N/A	1 other	No common toxin detected
14-5208	O179:H8	H8	H8	O179-manB	NR	NR	O179-wzx	N/A	4 others	No common toxin detected
14-5268	O71:H8	H8	H8	O71-qdtA	O71-wzx	N/A	N/A	N/A	stx1, hlyA, and 4 others	stx1, eae, hly

11-3188	O3:H9	H9	H9	O3-wclT	NR	O3-wzx	N/A	N/A	3 others	No common toxin detected
14-6184	Orough:H16	H16	H16	O74-galF*	NR	NR	NR	NR	stx2, hlyA subtilase, and 4 others	stx2c, hlyA
14-8461	O52:H45	H45	H45	O52-wzm	O52-wzx	N/A	N/A	N/A	stx1, hlyA, and 2 others	stx1
11-7440	O165:H25	H25	H25	O165-wcnt	O165-wzx	N/A	N/A	N/A	stx1, stx2, hlyA, intimin, and 7 others	Submitted by province as stx2+
15-0440	O95:H10	H10	H10	O95-rmlA	O87-wzt	N/A	N/A	N/A	2 others	No common toxin detected

*Regarded as common genes [1-4]; these genes represent the top hit from the database for the indicated O types except one O rough isolate, 14-6184. [A] Only important toxins [5] are shown, with the rest categorized as “others”: stx1, shiga-like toxin 1; stx2, shiga-like toxin 2; hlyA, hemolysin A. [B] Underlined genes were identified by WGS but not PCR; NR – No Results; N/A – Not applicable

Supplementary Table 3 Results of MS-H plus WGS-HOT analysis on 17 *E. coli* clinical strains designated "non-motile" (NM) by serotyping

Strain	Serotype	MS-H	H Antigen Gene Database	O antigen Gene Database					Toxin Gene Database[A]	Non-WGS based toxin tests
				Gene Cluster	Gene Pair 90%	Gene Pair 80%	Gene Pair 70%	Gene Pair 60%		
11-1100	O145:HNM	NI	H28	O145-wckD	O145-wzx	N/A	N/A	N/A	hlyA, intimin, and 5 others	hlyA
14-8460	O146:HNM	NI	H21	O146-rmlB<br>O46-gne*	O146-wzx	N/A	N/A	N/A	stx1, hlyA and 1 other	stx1
14-5211	O186:HNM	NI	H2	O123-fnlA<br>O186-fnlA	O123-wzx<br>O186-wzx	N/A	N/A	N/A	stx1, hlyA and 9 others	stx1, hlyA
11-5490	Orough: HNM	NI	H18	O176-ugd *<br>O44-galF*<br>O17-galF*<br>O77-galF*	NR	O77-wzx<br>O17-wzx	N/A	N/A	4 others	No common toxins detected
14-9143	O5:HNM	NI	H54	O5-wzx	O5-wzx	N/A	N/A	N/A	stx1, hlyA, intimin, and 8 others	stx1, intimin, hly
11-1264	O148:HNM	NI	H30	O148-wzy	O148-wzy	N/A	N/A	N/A	hlyA	hlyA
11-3615	O22:HNM	H33	H33	O83-wzz*	-	-	-	NR	6 others	No common toxin detected
14-7103	O21:HNM	H2	H27	O156-hisI*<br>O21-wclP	O21-wzy	N/A	N/A	N/A	2 others	No common toxins detected
14-5082	O26:HNM	H11	H11	O69-ugd*<br>O108-wzz*<br>O26-wbuA	O26-wzx	N/A	N/A	N/A	stx1, hlyA, intimin, and 9 others	stx1, intimin, hly
14-4602	O111:HNM	H8	H8	O111-manB	O111-wzx	N/A	N/A	N/A	stx1, stx2, hlyA, intimin, and 4 others	stx1, stx2, hlyA
14-4603	O111:HNM	H8	H8	O111-manB	O111-wzx	N/A	N/A	N/A	stx1, stx2, hlyA, intimin, and 5 others	stx1, stx2, hlyA
14-4604	O111:HNM	H8	H8	O111-wbdL	O111-wzx	N/A	N/A	N/A	stx1, stx2, hlyA, intimin, and 5 others	stx1, stx2, hlyA

11-5592	O111:HNM	H8	H8	O111-manB	O111-wzy	N/A	N/A	N/A	stx1, hlyA, intimin, and 5 others	Submitted by province as VT+
11-6320	O111:HNM	H8	H8	O111-wbdI	O111-wzy	N/A	N/A	N/A	stx1, stx2, hlyA, intimin, and 6 others	stx1, stx2, intimin, hlyA
14-8954	O111:HNM	H8	H8	O111-galF*	NR	NR	NR	NR	stx1, hlyA, intimin, and 3 others	stx1, hlyA
11-5807	O111:HNM	H8	H8	O111-wbdL	O111-wzy	N/A	N/A	N/A	stx1, hlyA, intimin, and 9 others	Submitted by province as VT+
11-2926	O157:HNM	H7	H7	O157-flc O86- ugd*	O157-wzx	N/A	N/A	N/A	stx1, stx2, hlyA, intimin, and 8 others	Submitted by province as VT+

*Common genes [1]. [A] Only important toxins [5] are shown, with the rest categorized as "others": stx1, shiga-like toxin 1; stx2, shiga-like toxin 2; hlyA, hemolysin A; N/A-Not applicable; NR-No results; NI-No identification.

Supplementary Table 4 Results of MS-H plus WGS-HOT analysis on five *E. coli* clinical strains designated "H undetermined" by serotyping

Strain	Serotype	MS-H	H Antigen Gene Database	O antigen Gene Database					Toxin Gene Database Search[A, B]	Non-WGS based toxin detection
				Gene Cluster	Gene Pair 90%	Gene Pair 80%	Gene Pair 70%	Gene Pair 60%		
14-7997	O107:Hund	H27	H27	O117-rmlC<br>O107-rmlC<br>O34-rmlB<br>O8-gne*	NR	NR	O107-wzy	NR	NR	No common toxin detected.
11-6574	O2:Hund	H7	H7	O14-wcaB<br>O2-ftdA<br>O50-rmlC	NR	NR	NR	NR	3 others	No common toxin detected.
14-8788	O13:Hund	H6	H6	O63-rmlA<br>O13-ugd*	O63-wzy	O63-wzy	O63-wzy	O63-wzy	stx2, intimin, and 2 others	stx2f, intimin,
11-6573	O16:Hund	H6	H6	O12-hisI*<br>O16-glf	O16-wzx	N/A	N/A	N/A	hlyA and 3 others	No common toxin detected
14-6742	O157:Hund	H27	H27	O153-gne*<br>O157-wbdR	NR	O157-wzx	N/A	N/A	3 others	No common toxin detected

*Common genes [1]. [A] Only important toxins [5] are shown, with the rest categorized as "others": stx1, shiga-like toxin 1; stx2, shiga-like toxin 2; hlyA, hemolysin A. [B] Underlined genes were identified by WGS but not PCR; N/A-Not applicable; NR-No results; Hund-H antigen undetermined

Supplementary Table 5 Results of MS-H plus WGS-HOT analysis on 21 *E. coli* clinical strains whose serotyping- and MS-H-designated H types were not in agreement

Strain	Serotype	MS-H	H antigen Gene Database	O antigen Gene Database					Toxin Gene Database Search[A, B]	Non-WGS based toxin detection
				Gene Cluster	Gene Pair 90%	Gene Pair 80%	Gene Pair 70%	Gene Pair 60%		
11-0357	O112ab:H21	H9	H9	O112ab-wfeI	NR	NR	NR	O112ab-wzx	1 other	No common toxin detected
14-4794	O174:H19	H7	H54	O8-wzm O174-wbcZ	O8-wzm	O8-wzm O174-wzy	N/A	N/A	1 other	No common toxin detected
11-5366	O16:H12	H6	H6	O16-rmlC O12-hisI*	O16-wzx	N/A	N/A	N/A	hlyA and 4 others	No common toxin detected
11-6008	Orough:H21	H2	H21	O10-galF*	NR	NR	NR	NR	stx1, stx2, hlyA, subtilase, and 5 others	Submitted by province as VT+
14-4323	O134:H1	H2	H31	O120-wzz*	NR	NR	O134-wzy	N/A	3 others	No common toxin detected
11-3186	O73:H29	H41	H41	O76-ugd* O44-gnd* O17-gnd* O77-manB O106-manB O73-manB	O77-wzx O17-wzx O44-wzx O73-wzx	N/A	N/A	N/A	1 other	No common toxin detected
11-3898	O120:H30	H12	H12	O153-gne* O69-hisI* O120-hisI*	NR	NR	NR	NR	3 others	No common toxin detected
11-3187	O153:H11	H26	H11	O37-galF* O90-wcaM O153-gne* O127-wcaM	NR	NR	NR	NR	stx2c, hlyA, intimin, and 6 others	hlyA

				O26-gnd*						
11-3195	O49:H10	H44	H10	O139-rmlA O49-vioA	O49-wzx	N/A	N/A	N/A	stx1, hlyA, and 5 others	stx2, hlyA
11-5769	Orough:H21	H54	H2	O153-gne*	NR	NR	NR	NR	2 others	No common toxin detected
11-5805	O26:H21	H11	H11	O69-ugd* O26-wbuA	O26-wzy	N/A	N/A	N/A	stx1, hlyA, intimin, and 15 others	Submitted by province as VT+
11-5130	O26:H21	H11	H11	O69-ugd* O26-rmlD	NR	O26-wzy	N/A	N/A	stx1, hlyA, intimin, and 8 others	Submitted by province as VT+
11-5593	O26:H21	H11	H11	O69-ugd* O26-rnl1	O26-wzy	N/A	N/A	N/A	stx1, hlyA, intimin, and 8 others	Submitted by province as VT+
14-5209	O103:H21	H4	H4	O103-wbtF	O103-wzx	N/A	N/A	N/A	3 others	No common toxin detected
11-4211	O103:H21	H2	H2	O10-galF* O103-wbtF	NR	O103-wzx	N/A	N/A	stx1, hlyA, intimin, and 4 others	Submitted by province as VT+
11-5367	O103:H21	H2	H2	O10-galF* O103-wbtC	O103-wzx	N/A	N/A	N/A	stx1, hlyA, intimin, and 5 others	stx1, intimin, hlyA
11-5595	O103:H21	H11	H11	O103-galE	O103-wzx	N/A	N/A	N/A	stx1, hlyA, intimin, and 12 others	Submitted by province as VT+
14-5114	O121:H1	H19	H19	O121-wbgF	O121-wzx	N/A	N/A	N/A	stx2, hlyA, intimin, and 9 others	stx2a, eae, hlyA
11-5594	O121:H1	H19	H19	O121-wbgF	O121-wzx	N/A	N/A	N/A	stx2, hlyA, intimin, and 9 others	Submitted by province as VT+
11-5597	O121:H1	H19	NR	O121-wbqE	O121-wzy	N/A	N/A	N/A	stx2, hlyA, and 3 others	Submitted by province as VT+
14-5069	O157:H7	H18	H7	O157-fcl O86- ugd*	O157-wzx	N/A	N/A	N/A	hlyA, intimin, and 6 others	hlyA

*Common genes [1]. [A] Only important toxins [5] are shown, with the rest categorized as “others”: stx1, shiga-like toxin 1; stx2, shiga-like toxin 2; hlyA, hemolysin A. [B] Underlined genes were identified by WGS but not PCR; N/A- Not applicable; NR-No results

Supplementary Table 6 Results of MS-H plus WGS-HOT analysis on *E. coli* clinical strains designated "O rough" by serotyping.

**Strain**	**Serotype**	**MS-H**	**H antigen Gene Database**	**O antigen Gene Database**					**Toxin Gene Database Search[A]**	**Non-WGS based toxin detection**
				**Gene Cluster**	**Gene Pair 90%**	**Gene Pair 80%**	**Gene Pair 70%**	**Gene Pair 60%**		
11-6008	O rough:H21	H2	H21	O10-galF*	NR	NR	NR	NR	stx1, stx2, hlyA, subtilase, and 5 others	Submitted by province as VT+
14-6184	O rough:H16	H16	H16	O74-galF*	NR	NR	NR	NR	stx2, hlyA subtilase, and 4 others	stx2c, hlyA
11-5490	O rough: HNM	H29	H18	O176-ugd* O44-galF* O17-galF* O77-galF*	NR	O77-wzx O17-wzx	N/A	N/A	4 others	No common toxin detected
11-5769	O rough:H21	H54	H2	O153-gne*	NR	NR	NR	NR	2 others	No common toxin detected

*Common genes [1]; [A] Only important toxins [5] are shown, with the rest were categorized as "others": stx1, shiga-like toxin 1; stx2, shiga-like toxin 2; hlyA, hemolysin A; N/A-Not applicable; NR-No results

Supplementary Table 7. Sequence comparison of two housekeeping genes*, galF* and *ugd*

7A, Sequence similarity (%) of *galF* gene sequences among three O antigen gene clusters

**Gene**	**Sequence Similarity (# identical nucleotides/total # of nucleotides)**		
galF	**010**	**074**	**0111**
**010**	--	96.08% (784/816)	82.42% (225/273)
**074**	96.08% (784/816)	--	80.96% (221/273)
**0111**	82.42% (225/273)	80.96% (221/273)	--

7B, Sequence similarity (%) of *ugd* gene sequences among three O antigen gene clusters

**Gene**	**Sequence Similarity (# identical nucleotides/total # of nucleotides)**		
ugd	**013**	**0108**	**0123**
**013**	--	96.92% (1131/1167)	96.83% (1130/1167)
**0108**	96.92% (1131/1167)	--	96.66% (1128/1167)
**0123**	96.83% (1130/1167)	96.66% (1128/1167)	--

Supplementary Table 8. Comparison of H typing results using the WGS-HOT and SeroTypeFinder platforms[A]

Batch	WGS code in IRIDA	Serotype	MS-H	WGS H typing	WGS O typing gene pairs at different coverage thresholds (%)				WGS gene cluster DB (90%)	CGE Serotype Finder[B]
					90	80	70	60		
1	14-5082	O26:HNM	H11	H11	O26-wzx	N/A	N/A	N/A	O26 wbuA	O26:H11
	14-5114	O121:H1	H19	H19	O121 wzx	N/A	N/A	N/A	O121 wbgF	O121:H19
	14-5209	O103:H21	H4	H4	O103 wzx	N/A	N/A	N/A	O103 wbtF	O103:H4
	E32511*	O157:HNM	H7	H7	O157wzx	N/A	N/A	N/A	O157 flc	O157:H7
2	14-5069	O157:H7	NO	H7	O157 wzx	N/A	N/A	N/A	O157 wbdR	O157:H7
	11-5595	O103:H21	H11	H11	O103 wzx	N/A	N/A	N/A	O103 galE	O?:H11
	14-6184	Orough:H16	H16	H16	NR	NR	NR	NR	NR	O?:H16
	EDL933*	O157:H7	H7	H7	NR	O157 wzx	N/A	N/A	O157 wbdQ	O?:H7
3	H19*	O26:H11	NP	H11	O26 wzy	N/A	N/A	N/A	O26 wbuA	O26:H11
	86-704*	O15:H27	NP	H27	NR	NR	NR	NR	O15 wzy	O15:H27
	90-2380*	O157:H7	H7	H7	O157 wzx	N/A	N/A	N/A	O157 wbdO	O157:H7
	25922*	O6:H1	NP	H54	O6 wzx	N/A	N/A	N/A	O6 wzx	O6:H1

4	11-5591	O45:H2	H2	H2	O45 wzx	N/A	N/A	N/A	O45 wbhU	O45:H2
	14-6742	O157:HNM	H27	H27	NR	O157 wzx	N/A	N/A	O157 wbdR	O?:H27
	14-6526	O175:H28	H28	H11	NR	O26 wzx	N/A	N/A	O26 wbuA	O26:H11
	14-4150	O181:H4	H4	H4	NR	NR	NR	NR	O181 fnlA	O181:H4
5	11-7434	O121:H19	NR	H19 @60% coverage	NR	NR	NR	NR	O121 wfbD	O?:H19
	11-7436	O103:H25	H25	H25 @70% coverage	NR	NR	NR	NR	O103 wbtA	O?:H25
	93-2003*	O157:H7	NP	H7	NR	NR	NR	O157 wzx	O157 fcl	O157:H7
	14-6184[#]	Orough:H16	H16	H16	NR	NR	NR	NR	NR	O?:H16

[A] Samples were randomly chosen for comparison from 5 batches of WGS-HOT sample submission. [B] SeroTypeFinder search parameters were set as follows: threshold for % ID = 30%, minimum length = 20% on assembled genome contigs. * Reference strain; [#] Isolate underwent repeat testing; N/A, Not applicable; NR, No result; NP, Not performed; NM, Non-motile; O?, O antigen could not be identified

References related to the Supplementary Tables:

1. **DebRoy, C., E. Roberts, and P. M. Fratamico.** 2011. Detection of O antigens in *Escherichia coli*. Anim. Health. Res. Rev. **12:**169-185.

2. **Wang, W., A. V. Perepelov, L. Feng, S. D. Shevelev, Q. Wang, S. N. Senchenkova, W. Han, Y. Li, A. S. Shashkov, Y. A. Knirel, P. R. Reeves, and L. Wang.** 2007. A group of *Escherichia coli* and *Salmonella enterica* O antigens sharing a common backbone structure. Microbiology. **153:**2159-2167.

3. **Wang, Q., A. V. Perepelov, L. Wen, A. S. Shashkov, X. Wang, X. Guo, Y. A. Knirel, and L. Wang.** 2012. Identification of the two glycosyltransferase genes responsible for the difference between *Escherichia coli* O107 and O117 O-antigens. Glycobiology. **22:**281-287.

4. **Feng, L., W. Han, Q. Wang, D. A. Bastin, and L. Wang.** 2005. Characterization of *Escherichia coli* O86 O-antigen gene cluster and identification of O86-specific genes. Vet. Microbiol. **106:**241-248.

5. **Wang, G., C. G. Clark, and F. G. Rodgers.** 2002. Detection in *Escherichia coli* of the genes encoding the major virulence factors, the genes defining the O157:H7 serotype, and components of the type 2 Shiga toxin family by multiplex PCR. J. Clin. Microbiol. **40:**3613-3619.
