# Supplementary material for: Phenotypic H-Antigen Typing by Mass Spectrometry Combined with Genetic Typing of H Antigens, O Antigens, and Toxins by Whole-Genome Sequencing Enhances Identification of Escherichia coli Isolates
Source: J Clin Microbiol. 2016 Jul 25;54(8):2162–8. doi: 10.1128/JCM.00422-16 (PMC4963523; doi:10.1128/JCM.00422-16)
Supplement: Supplemental material [file JCM.00422-16_zjm999095110so2.pdf]

Supplementary Text

E. coli reference strains and clinical isolates

All *E. coli* reference strains were obtained from stocks at the International Organization for Standardization (ISO)-certified National Enterics Reference Services Centre at the National Microbiology Laboratory in Winnipeg, Manitoba, Canada. Clinical isolates were obtained from five Canadian provincial laboratories: AB, MB, NL, NS, and QC.

Flagella extraction, digestion, and preparation for mass spectrometry detection

All *E. coli* strains were grown overnight at 37°C ± 2°C on tryptic soy agar plates with 5% sheep blood. For standard flagella extraction and on-filter digestion, a loop-full of culture was diluted in 1 ml of water and gently suspended using a pipette tip. The samples were then vortexed (Vortex Genie-2, Scientific Industries) at maximum speed for three cycles of 20 sec and 1 min rest, and centrifuged at 16,000 x g for 20 min in a bench top centrifuge (Eppendorf Centrifuge 5417C). The supernatant was collected with a 1 ml syringe (without a needle) and passed through a 13 mm diameter filter with a pore size of 0.20 µm (Acrodisc, PALL). The filters were gently washed with approximately 3 ml of water using a 10 ml syringe, and flushed with air using a 1 ml syringe. Next, 100 µl of trypsin (MS grade, Pierce; 100 µg/ml in 100 mM ammonium bicarbonate) was applied to the filter and the samples were placed at 37°C ± 2°C for 2 hours to allow digestion to occur. The filters were washed with approximately 600 µl (12 drops) of water and flushed with air to collect the digest. Twenty five µl of the collected digest was then mixed with 25 µl of Buffer A (0.2% formic acid with 1% ACN), and 10 µl of the mixture was loaded for mass spectrometry detection. Nano-LC (Proxeon) separation was run at 300 nl/min with a 45 min acetonitrile gradient from 5-36% followed by a 5 min flush with 95%

acetonitrile and 10 min equilibration with Buffer A. Mass spectrometry data were collected from an LTQ-Orbitrap XL system (ThermoFisher) with a data-dependent acquisition method for peptide ion scanning and fragmentation. The mass spectrometry data were searched with Mascot 2.3 (Matrix Science) against our custom flagella protein database, which was updated in December 2014. Search parameters included 30 ppm mass error tolerance, 2 missed cleavages for trypsin digestion with no fixed modifications of proteins, and possible modifications of methionine oxidation and glutamine and asparagine deamination. The following rules were then established and used to assign an H type: (A) a minimum emPAI value of 1 was necessary; (B) the top hit should have an emPAI value at least twice that of the previous adjacent blank run when the emPAI value of the previous blank run exceeded 1 due to carryover; (C) repeated jigsaw cleanups and LC-MS/MS analyses of samples were performed after blank runs showing emPAI values above 1; and (D) if the adjacent previous blank run had no significant flagellin identified (less than two specific peptides) and the emPAI value of the subsequent sample run was 0.10-0.99 due to low flagella production from "sluggish" isolates, repeat testing should be performed with a higher sample amount to obtain an emPAI value equal to or above 1.

DNA extraction and whole genome sequencing (WGS)

All reagents were obtained from Epicentre Metagenomic DNA Isolation kits unless otherwise noted. Strains were obtained from freezer stocks and incubated overnight at 37°C ± 2°C on tryptic soy agar (TSA) plates containing 5% sheep blood, and then cultured a second time onto new TSA 5% sheep blood plates and incubated overnight again at 37°C ± 2°C. The culture from the second day was used for DNA extraction. Four average sized colonies were taken from the plate using the sides of a loop and then swirled into a microcentrifuge tube containing 300 µl of Tris-EDTA (TE) buffer, 2 µl of Ready-Lyse Lysozyme, and 2 µl RNaseA (5µg/µL). The

samples were then mixed thoroughly by pipetting to ensure a homogenous suspension and incubated at 37°C ± 2°C for a minimum of 30 min. Three hundred microliters of Meta-Lysis Solution (2x) and 3 µl of Proteinase K (50 µg/µl) was added to each sample, which were then incubated at 65°C ± 2°C for 15 min. The samples were placed at 4°C for 7 min to cool to room temperature and placed on ice for 5 min. Once cooled, 350 µl of MPC Protein Precipitation Reagent was added to each sample and mixed vigorously by vortexing for approximately 10 sec or until the suspension was homogenous. The mixture was centrifuged for 10 min at 14,000 x g at 4°C in order to pellet the debris. The supernatant was immediately transferred to a clean microcentrifuge tube and the samples centrifuged at 16,000 x g at 4°C. The supernatant was again transferred to a clean microcentrifuge tube and 4 µl of RNaseA (100mg/ml, Qiagen) was added to each sample and vortexed for a few seconds to mix. The samples were then incubated at 37°C ± 2°C for 5 min. Six hundred microliters of isopropanol was added to each sample, the tubes were inverted multiple times to mix, and then placed at 4°C for a minimum of 30 min to allow the DNA to precipitate. The DNA was then pelleted by centrifugation for 10 min at 14,000 x g at 4°C and the supernatant carefully removed so as not to disturb the pellet. Five hundred microliters of pre-chilled 70% ethanol was added to the pellet, and the samples were centrifuged again for 5 min at 14,000 x g at 4°C. The ethanol was carefully removed from the tube, and the samples were pulse centrifuged to ensure residual liquid was also removed. The DNA pellets were then air dried for 8 min at room temperature and re-suspended in 50 µl of 1x Tris buffer (10 mM Tris, EB buffer or elution buffer, pH 8.5, Qiagen). Optical density ratios at 260/280 nm and 260/230 nm were measured using a Nanodrop (ThermoFisher) to ensure the samples were free from major contamination. Agarose gel electrophoresis was also performed by loading a 1 µl aliquot of each sample into a 1% gel along with Fosmid Control DNA (40kb, 100ng/µL). The gel

ran at 120V for 30 minutes, and was used to determine that there was no RNA contamination in the sample nor had the DNA been sheared during the extraction process. The final quantitation of DNA was performed using a Qubit broad range kit (Invitrogen) and the DNA equilibrated with EB buffer in concentrations ranging from 10-50 ng/µL. WGS was performed using a Nextera XT DNA Sample Preparation Kit (Illumina) and data acquired by 300 bp paired-end sequencing on the Illumina MiSeq using the MiSeq Reagent Kit V2 (600 cycles). An *in silico* analysis was performed on the sequences of each isolate, whereby data were assembled into contigs using SPAdes Assembler (v2.5.1). Contig output was then searched against a custom database containing *E. coli* H antigen, O antigen, and toxin sequences (in FASTA format) extracted from the NCBInr database. For O antigens, two databases were created: a gene pair database containing *wzx*/*wzy* or *wzm*/*wzt* genes pairs only, or a gene cluster database containing all O antigen-related genes. Galaxy software was used to perform the database search. The parameters of the WGS workflow called for a minimum of 90% coverage between the database and WGS data, as well as a maximum divergence of 5 in order for a region to be considered a match and be displayed in the list of results. In addition, various coverages (60-90%) were used for the O antigen gene pair database search. The top match was determined to be the result with the lowest divergence.
